# Anti-inflammatory effect of supercritical extract and its constituents from Ishige okamurae

**DOI:** 10.17179/excli2016-337

**Published:** 2016-06-28

**Authors:** Eun-Yi Ko, Weon-Jong Yoon, Hae-Won Lee, Soo-Jin Heo, Young-Hwan Ko, I.P. Shanura Fernando, Kichul Cho, Chi-Heon Lee, Sung-Pyo Hur, Su-Hyeon Cho, Ginnae Ahn, Daekyung Kim, Kil-Nam Kim

**Affiliations:** 1 Jeju Center, Korea Basic Science Institute (KBSI), Jeju 690-140, Republic of Korea; 2 Department of Marine Life Science, Jeju National University, Jeju 690-756, Republic of Korea; 3 Jeju Biodiversity Research Institute, Jeju Technopark, Jeju, 699-943, Republic of Korea; 4 World Institute of Kimchi, Gwangju 503-360, Republic of Korea; 5 Jeju International Marine Science Center for Research & Education, Korea Institute of Ocean Science & Technology (KIOST), Jeju, 63349, Republic of Korea; 6 Department of Food Bioengineering, Jeju National University, Jeju 690-756, Republic of Korea; 7 Department of Marine Bio-Food Sciences, Chonnam National University, Yeosu 550-74, Republic Korea; 8 Department of Marine Biotechnology, University of Science and Technology, Daejeon 305-350, Republic of Korea

**Keywords:** supercritical fluid extraction, linoleic acid, Ishige okamurae, anti-inflammatory, NF-kappaB

## Abstract

The anti-inflammatory properties of the supercritical fluid extract of *Ishige okamurae* (SFEIO) on lipopolysaccharide (LPS)-stimulated murine RAW 264.7 macrophages. The lipid profile of the SFEIO, reviled the presence of palmitic acid (220.2 mg/g), linoleic acid (168.0 mg/g), and oleic acid (123.0 mg/g). SFEIO was found to exert it's anti-inflammatory effects through inhibiting nitric oxide (NO), prostaglandin E_2_ (PGE_2_), inducible nitric oxide synthase (iNOS), cyclooxygenase-2 (COX-2), tumor necrosis factor (TNF)-α, interleukin (IL)-1β, and IL-6 production in LPS-stimulated RAW 264.7 cells, without inducing cytotoxicity. SFEIO did not effect on the LPS-induced p38 kinase phosphorylation, whereas it attenuated the extracellular-related signaling kinase (ERK) and c-Jun N-terminal kinase (JNK) phosphorylation. Furthermore, SFEIO inhibited the LPS-induced IκB-α degradation and p50 NF-κB activation. These results suggest that SFEIO exerts its anti-inflammatory effects in LPS-activated RAW 264.7 cells by down-regulating the activation of ERK, JNK, and NF-κB.

## Introduction

Inflammatory diseases have become one of the major health issues in many parts of the world having a considerable influence on the physiological well-being and health care costs. Inflammation can be defined as a non-specific protective response of the body to defend the host from pathogens, toxins, and local injuries. Inflammatory reactions are typically characterized by the redness, swelling, heat, and pain (Medzhitov, 2008[23]). Pathogen- and host-derived molecules (lipopolysaccharide (LPS) and interferon-γ (IFN-γ)) can stimulate macrophages, thereby upregulate inflammatory mediators. These includes nitric oxide (NO), prostaglandin E_2_ (PGE_2_), inducible nitric oxide synthase (iNOS), and cyclooxygenase-2 (COX-2) (Korhonen et al., 2005[19]). These are considered as some of the major inflammatory mediators that regulate essential steps in inflammatory signaling pathways. Therefore, inhibiting the production of these inflammatory mediators is an important target for the anti-inflammatory drugs to treat inflammatory diseases. 

Among a countless number of extraction methods in natural product chemistry, carbon dioxide (CO_2_) has gained much attention as an extraction solvent for natural products. It is a non-toxic, readily available and non-explosive solvent that could easy to remove from the extract. The extraction can be achieved by the supercritical fluid extraction (SFE) methodology which utilizes low temperatures, resulting in less deterioration of thermally labile components. In addition, SFE with CO_2_ can ensure that the active ingredients undergo minimal alterations, preserving their curative properties (Nasser et al., 1992[25]; Oszagyán et al., 1996[28]). Thus, SFE with CO_2_ can be considered as an effective extraction method to obtain bio-actives. 

Marine algae have intensively been studied for their vast diversity of physiological effects, including whitening, anti-inflammatory, anti-cancer, and anti-obesity activity (El Gamal, 2010[11]; Li et al., 2011[21]). *Ishige okamurae* is an edible brown alga found along the coast of Jeju Island in South Korea. Several studies have reported the biological benefits of *I. okamurae* extracts, including its inhibitory effects on HIV (Ahn et al., 2006[1]) and bacterial phospholipase A2 activity (Cho et al., 2008[7]), anti-inflammatory effects (Kim et al., 2010[16], 2009[18]), and anti-oxidant effects in free radical-mediated oxidative systems (Zou et al., 2008[39]). However, the anti-inflammatory effects of the SFE from *I. okamurae* (SFEIO) remain unexplored. The main objectives of the current study were to evaluate the fatty acid composition of SFEIO and to investigate its anti-inflammatory activity in LPS-activated murine macrophage (RAW 264.7) cells.

## Material and Methods

### Reagents

Fetal bovine serum (FBS), Dulbecco's modified Eagle's medium (DMEM), antibiotics (penicillin and streptomycin were obtained from Invitrogen-Gibco (Grand Island,NY). Dimethyl sulfoxide (DMSO) , lipopolysaccharide (LPS; from *Escherichia coli *strain) and phosphate buffered saline (PBS) were purchased from Sigma Chemical Co(St. Louis, MO, USA). The ELISA kits for TNF-α, PGE_2_, IL-1β, and IL-6 were obtained from BD Biosciences (San Diego, CA, USA) and R&D Systems, Inc. (St. Louis, MO, USA). Anti-bodies that includes: iNOS, COX-1, COX-2, IκB-α, ERK, JNK, p38, p-ERK, p-JNK, p-p38 and β-actin were purchased from Cell Signaling Technology (Beverly, MA, USA). The secondary antibodies were obtained from Cell Signaling Technology.

### Supercritical fluid extraction of I. okamurae

The marine alga *I. okamurae* was collected off the Jeju Island, South Korea in March 2011 and was identified by Dr. Dong-Sam Kim. The sample was dually washed using tap water to remove salt, epiphytes, and other debris attached to the surface, rinsed carefully in fresh water, and dried at room temperature for 2 weeks. The dried sample was ground into a powder prior to extraction. A supercritical extraction system (ILSHIN Autoclave Co., Daejueon, Korea) with a 500 mL extraction cell was used to perform SFE on *I. okamurae. *The extraction cell was filled with 300 g of powdered *I. okamurae*. The extraction was conducted at 40° C for 2 h, providing an extraction pressure of 400 bars. 

### Fatty acid analysis 

Fatty acid profile of the sample was determined using a modified fatty acid methyl ester (FAME) method (Morrison and Smith, 1964[24]). Initially, 18.38 mg of the sample was weighed into a 30 mm × 110 mm Pyrex tubes fitted with a Teflon-lined cap. Undecanoic acid in dichloromethane was added as the internal standard. A volume of 1.5 mL of 0.5 N NaOH in methanol was introduced to the tubes, vortexed and incubated at 100° C for 5 min. After the incubation, 2 mL of boron trifluoride (14 %) in methanol was introduced and incubated at 100° C for 5 min. Tubes were then removed and cooled to room temperature before adding hexane (1 mL) to extract the FAMEs. A solution of saturated sodium chloride (5 mL) was added, and the mixture was shaken briefly and kept undisturbed allowing the phase separation. The hexane phase was then transferred to a conical tube. The tube remains were again partitioned with hexane (1 mL). The hexane phase was filtered through a silicon-treated Whatman filter paper to remove any residual water.

A Shimadzu GC-2010 plus gas chromatographic (GC) system, equipped with an SP-2560 fused silica capillary column (100 m × 0.25 mm i.d. and 0.2 μm film thickness) (Supelco, Inc., Bellefonte, PA, USA) coupled to a flame ionization detector (FID) was used to analyze the FAMEs. Helium was used as carrier gas at a continuous flow rate of 2.41 mL/min, given a head pressure of 403.1 kPa and a 1:100 split ratio. Both the injector and detector temperatures were held at 260° C. The oven temperature was increased from 100 to 240° C at a rate of 4° C/min and then held constant for 20 min. GC retention time and the fragmentation patterns of FAMEs were used to identify the components. Quantitative measurements were taken using their response factors calculated by comparing the peak areas of the standards to the internal standard, undecanoic acid. 

### Cell culture

Murine macrophage RAW 264.7 cells were obtained from the American Type Culture Collection (ATCC, Manassas, VA, USA). The cells were maintained at 5 × 10^5^ cells/mL in DMEM medium supplemented with 10 % FBS, Each 100 units/mL of antibiotics, penicillin and streptomycin were incubated at 37° C in a humidified atmosphere containing 5 % CO_2_.

### Determination of NO production

The nitrite concentration in the medium was measured as an indicator of NO production using the Griess reagent. For this, RAW 264.7 cells (2 × 10^5^ cells/ml) were pre-incubated for 24 hours at 37° C following the treatment of SFEIO and LPS (1ug/ml), the quantity of nitrite accumulated in the culture medium was measured as an indicator of NO production. The evaluation was done by mixing equal volumes of culture medium and Griess reagent (1 % sulfanilamide in 5 % phosphoric acid and 0.1 % naphtylethylenediamine dihydrochloride in distilled water). The mixture was allowed to reach the equilibrium at room temperature for 10 min, and the absorbance was determined at 540 nm with a microplate reader . Fresh culture medium was used as the blank in every experiment. Nitrite levels in samples were determined using a standard sodium nitrite curve.

### Lactic dehydrogenase (LDH) cytotoxicity assay

Sophocarpine-induced cytotoxicity was measured using the cytotoxicity detection kit (LDH) (Nanjing Jiancheng Co.). RAW 264.7 cells (1.5 × 10^5^ cells/ml) were plated in 96 well plates and pre-incubated for 24 hours. Then the samples and LPS (1 μg/ml) were treated to the plates following an incubation period of 24 h at 37° C. The medium was then collected, and the amount of LDH released by cells was determined using an assay kit according to the manufacturer's instructions. Finally, the absorbance of each well was measured at 490 nm using a microplate reader.

### Determination of pro-inflammatory cytokines (TNF-α, IL-1β, and IL-6) and prostaglandin E_2_ (PGE_2_) production

Macrophages were cultured in 24 well plates allowing 24 hours of post-incubation and treated with different concentrations of SFEIO (SFE from *I. okamurae)* followed by LPS (1 μg/ml) and further incubated for another 24 hours at 37° C. Finally, the cytokines and PGE_2_ concentrations in the culture medium were quantified using a competitive enzyme immunoassay kit (R&D Systems, Minneapolis, MN, USA) according to the manufacturer's instructions. 

### Immunoblotting

RAW 264.7 macrophages were cultured at a concentration of 5 × 10^5^ cells per dish and incubated at 37° C for 24 h. Then the cells were treated with different concentrations of SFEIO (SFE from *I. okamurae)* and incubated for 24 h. The cells were lysed using a RIPA buffer [50 mM Tris-HCl pH 7.4, 150 mM NaCl, 1 mM EDTA, 1 % Triton X-100, 1 % sodium deoxycholate, 0.1 % sodium dodecyl sulfate (SDS)]. Protein concentrations were determined using a Bio-Rad protein assay kit (Bio-Rad, CA, USA) using bovine serum albumin (BSA) as the calibration standard. Cell lysates were electrophoresed in SDS-polyacrylamide gels (8-12 %), and the separated proteins were transferred to PVDF membranes (Bio-Rad). The immunoblot was kept in the blocking solution (Tris-buffered saline/Tween 20, TBST) containing 5 % skim milk (w/v) for 3 h under gentle shaking conditions at room temperature. Then the membranes were incubated with primary antibodies, iNOS,COX-2, IκB-α, p-p50, p-ERK, ERK, p-JNK and JNK(cell signaling; 1:1000) in 5 % BSA in TBST for 24h at 4° C. After the removal of the primary antibodies, the membranes were washed three times with TBST buffer at room temperature, and incubated with peroxidase-conjugated secondary antibodies in 5 % BSA in TBST (diluted 1:5000; Cell signaling) for 2 h at room temperature. Following the addition, signals were developed using ECL western blotting detection kit exposed to Biorad Chemidac system.

### Statistical analysis

All data in this study are expressed as means ± S.D. Significant differences among the groups were determined using the unpaired Student's *t*-test. A value of *p < 0.05 was considered to be statistically significant.

## Results

### Fatty acid profile of SFEIO 

**Figure 1:**
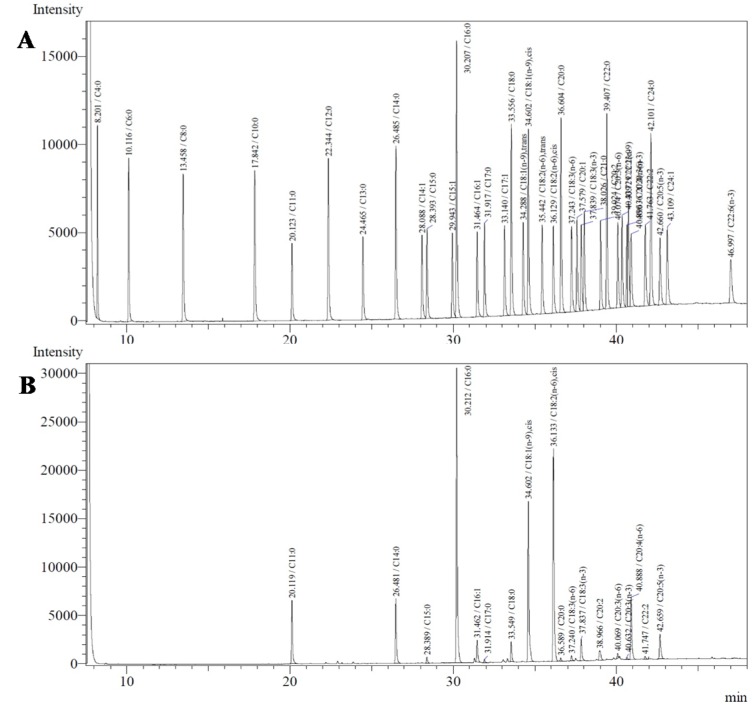
Chromatogram of the FAME of the (A) standard (C4:0-C22:6 methyl esters) and (B) SFEIO

Table 1(Tab. 1), summarize the results of the fatty acid analysis for the SFEIO and the Figure 1(Fig. 1) represents the corresponding chromatograms for the fatty acid composition in SFEIO and the used standard. A total of 37 fatty acids were investigated, and 17 of them were identified in SFEIO. Palmitic acid (30.2 %) was the most abundant constituent in SFEIO, followed by linoleic acid (23.1 %) and oleic acid (16.9 %). Total fatty acid content in SFEIO calculated as the sum of the individual fatty acids, was 728.4 mg/g extract wt; the total saturated and the unsaturated fatty acid content was 292.4 and 436.0 mg/g extract wt, respectively contributing to 40.1 % and 59.9 % of the total fatty acid content. 

### Effect of SFEIO on production of NO and PGE_2_ in LPS-stimulated RAW 264.7 cells

**Figure 2:**
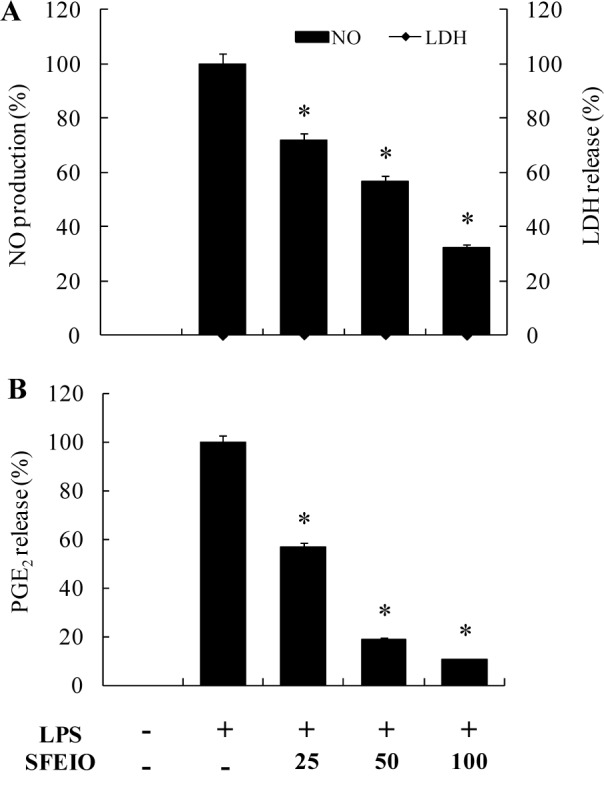
Effects of SFEIO on (A) NO and (B) PGE_2_ production in LPS-induced RAW 264.7 cells. Cells stimulated with LPS (1 μg/ml) in the presence of SFEIO (25, 50, and 100 μg/ml) for 24 h at 37° C. (A) Cytotoxicity was determined using the LDH method. Values are expressed as means ± S.D. of triplicate experiments. *P < 0.05 indicate significant differences from the LPS-stimulated group.

The effect of SFEIO on LPS-stimulated NO and PGE_2_ production in RAW 264.7 cells were assessed by treating the cells with LPS except for the control following the treatment of SFEIO (25, 50, or 100 μg/mL). Nitrite and PGE_2_ levels in unstimulated RAW 264.7 cells were negligible; however, their levels were increased substantially following the LPS stimulation (Figure 2(Fig. 2)). Treatment of RAW 264.7 cells with SFEIO significantly and dose-dependently inhibited LPS-stimulated NO and PGE_2_ production (Figure 2(Fig. 2)). To examine whether the inhibitory effects of SFEIO on production of pro-inflammatory mediators in LPS-stimulated RAW 264.7 cells were due to cytotoxicity (Figure 2A(Fig. 2)). Based on the results, SFEIO did not induce cytotoxicity in RAW 264.7 cells at the evaluated concentrations. 

### Effects of SFEIO on iNOS and COX-2 protein expression in LPS-stimulated RAW 264.7 cells

**Figure 3:**
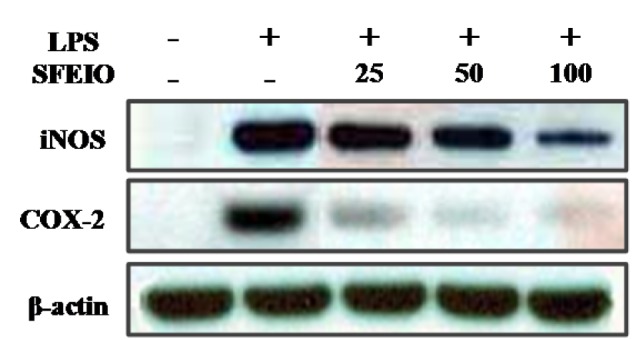
Effects of SFEIO on LPS-induced iNOS and COX-2 protein expressions in RAW 264.7 cells. Cells stimulated with LPS (1 μg/ml) in the presence of SFEIO (25, 50, and 100 μg/ml) for 24 h at 37° C. iNOS and COX-2 protein level were determined via Western blotting.

iNOS and COX-2 are two of the most important signaling molecules that regulate inflammatory reactions. Western blot analysis was used to determine the effects of SFEIO on iNOS and COX-2 protein expression in RAW 264.7 cells upon treatment with different concentrations of SFEIO (25, 50, or 100 μg/mL) as SFEIO was found to inhibits NO and PGE_2_ production in LPS-stimulated RAW 264.7 cells. According to the results, iNOS and COX-2 protein expression were almost undetectable in unstimulated RAW 264.7 cells. Whereas, treatment with 1.0 μg/mL LPS significantly increased the iNOS and COX-2 protein expression. However, the treatment of SFEIO significantly and dose-dependently downregulated the iNOS and COX-2 protein expression in LPS-stimulated RAW 264.7 cells (Figure 3(Fig. 3)). Therefore, the inhibition of NO and PGE_2_ production in LPS-stimulated RAW 264.7 cells is attributed to the inhibition of iNOS and COX-2 expression.

### Effects of SFEIO on pro-inflammatory cytokine expression in LPS-stimulated RAW 264.7 cells

**Figure 4:**
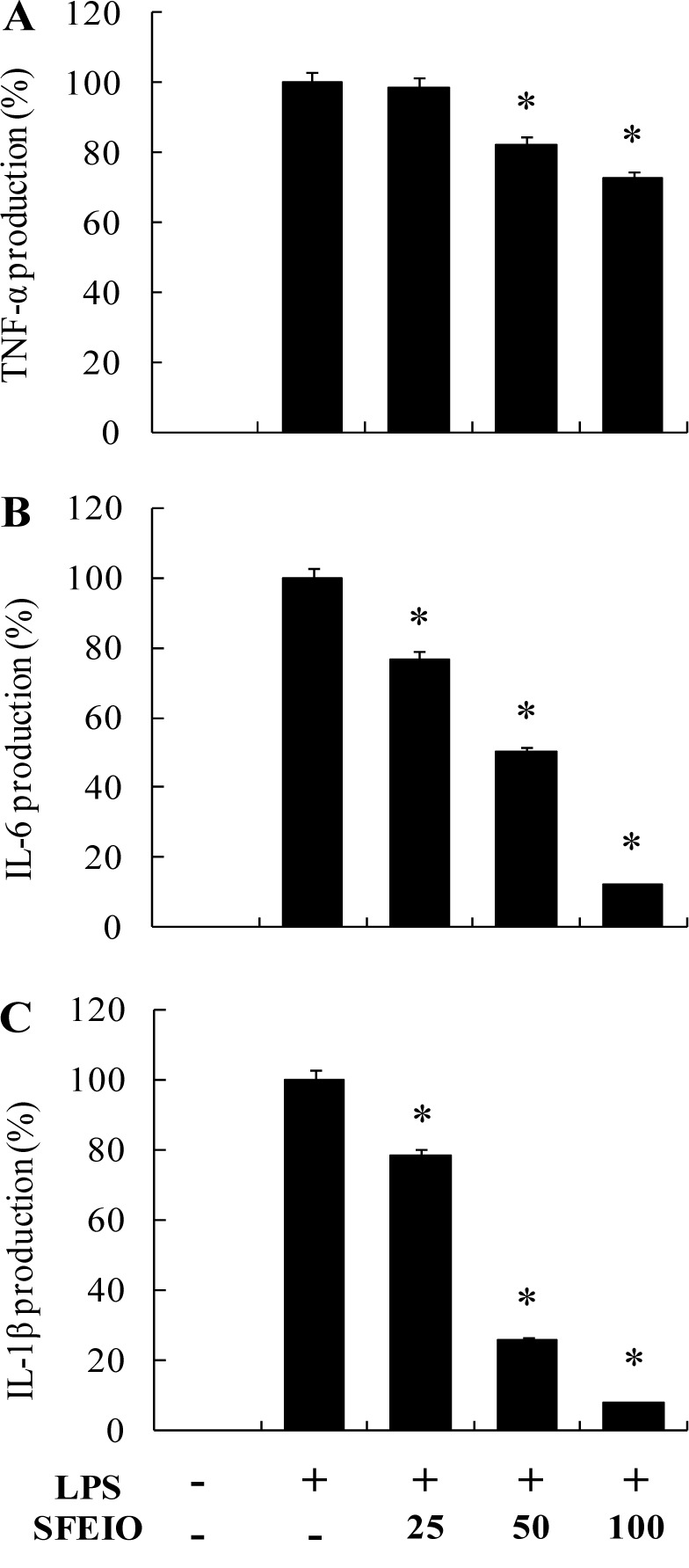
Inhibitory effects of SFEIO on the pro-inflammatory cytokine production in RAW 264.7 cells. The production of (A) TNF-α, (B) IL-6, and (C) IL-1β were assayed in the culture medium of cells stimulated with LPS (1 μg/ml) for 24 h in the presence of SFEIO (25, 50, and 100 μg/ml). Supernatants were collected, and the TNF-α, IL-6, and IL-1β concentration in the supernatants were determined by ELISA. Values are expressed as means ± S.D. of triplicate experiments. *P < 0.05.

Tumor necrosis factor (TNF)-α, interleukin (IL)-1β, and IL-6 are some of the pro-inflammatory cytokines that play important roles in immune responses variety of inflammatory stimuli (Bertolini et al., 2001[4]; Lind, 2003[22]). Therefore, the inhibitory effects of SFEIO on LPS-stimulated TNF-α, IL-1β, and IL-6 production were dually examined to identify the expression levels. These levels were measured in culture supernatants using enzyme-linked immunosorbent assay (ELISA) kits. Treatment of RAW 264.7 cells with LPS significantly up-regulated the cytokine production, compared to the non-treated group. However, TNF-α, IL-1β, and IL-6 levels were significantly and dose-dependently reduced relative to the positive control treated with LPS (Figure 4(Fig. 4)).

### Effect of SEIO on LPS-induced NF-kappaB and mitogen-activated protein kinase (MAPK) activation

**Figure 5:**
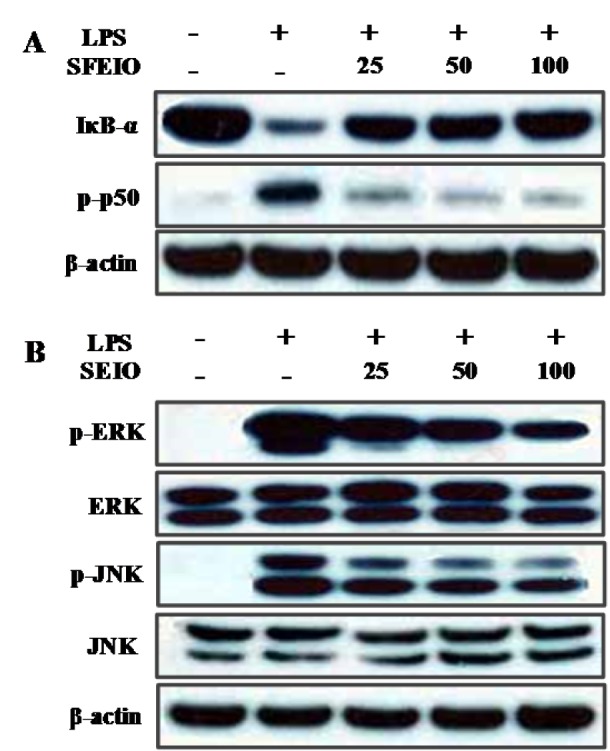
Inhibitory effects of SFEIO on LPS-induced phosphorylation of (A) IκB-α, NF-κB p50, (B) JNK, and ERK. Cells were treated for 15 min with LPS (1 μg/ml) alone or with LPS (1 μg/ml) coupled with different concentrations (025, 50, and 100 μg/ml) of SFEIO. Cell lysates were extracted, and protein levels of p- IκB-α, p-p50 p-ERK, ERK, p-JNK, and JNK were analyzed by Western blot.

NF-κB plays an important role in the transcription of pro-inflammatory mediators following LPS stimulation (Henkel et al., 1993[13]; Rhee et al., 2007[30]). Therefore, western blot analysis was performed to assess the effect of SFEIO on IκBα and p50 NF-κB phosphorylation. As shown in Figure 4A(Fig. 4), treatment with LPS induced IκBα degradation, which was significantly blocked by SFEIO. SFEIO also dose-dependently inhibited LPS-induced p50 NF-κB activation (Figure 5A(Fig. 5)). 

MAPK are known to regulate the expression of inflammatory enzymes and cytokines (Hommes et al., 2003[15]). To determine whether the inhibition of inflammation was mediated via the MAPK pathway, phosphorylation of p38, extracellular-signaling related kinase 1/2 (ERK1/2), and c-Jun N-terminal kinase (JNK) in LPS-induced cells were examined by western blot. As shown in Figure 4B(Fig. 4), LPS-stimulation markedly increased p38, ERK1/2, and JNK phosphorylation. However, SFEIO significantly inhibited LPS-stimulated ERK and JNK phosphorylation. Importantly, the levels of non-phosphorylated MAPK isoforms did not vary significantly among groups (Figure 5B(Fig. 5)). In contrast, SFEIO treatment did not affect the LPS-stimulated p38 protein phosphorylation (data not shown).

## Discussion

SFE has several advantages over conventional solvent extraction, as it shows increased selectivity, decreased time consumption, and it is environmentally friendly (Shu et al., 2004[33]). However, *I. okamurae *has not previously been extracted using SFE for bioactivity screenings. Thus, we investigated the anti-inflammatory effects and fatty acid constituents of *I. okamurae* extracts by using SFE technology.

Fatty acids from seaweeds can be obtained by using SFE as extensively reported (Cheung, 1999[6]; Herrero et al., 2006[14]). Therefore, we first measured the fatty acids composition of SFEIO. The major fatty acid components in the extract were palmitic acid (30.2 %), linoleic acid (23.1 %) and oleic acid (16.9 %). Unsaturated fatty acids contributed to 59.9 % of the total fatty acid content. Fatty acids such as phospholipids play a key role in maintaining the structural integrity of the cell membrane and play important roles in maintaining various cellular functions including metabolism and immune responses (Basu et al., 2006[3]; Cabral, 2005[5]; Natali et al., 2007[26]). The dietary intake of mono- and polyunsaturated fatty acids is associated with reduced risk of cardiovascular diseases including hypertension and atherosclerosis (Basu et al., 2006[3]). Polyunsaturated linoleic acid inhibits COX-1 and COX-2 in vitro (Ciaraldi et al., 2002[8]; Ringbom et al., 2001[31]) and decreases the expression levels of NF-κB and TNF-α genes in human THP-1 monocytic cells, leading to a decreased production of IL-6, IL-1β and TNF-α levels (Zhao et al., 2005[38]). Recently, the monounsaturated oleic acid was found to inhibit LPS-induced iNOS and COX-2 expression through blocking p38 and NF-κB signaling pathways in BV2 murine microglia (Oh et al., 2009[27]). Palmitic acid has been shown to trigger inflammation in cultured human macrophages (Laine et al., 2007[20]) and increasing TNF-α and IL-6 production via p38 and ERK activation in astrocytes cells (Gupta et al., 2012[12]). However, ω-3 fatty acids could act in a dose-dependent manner preventing the palmitic acid-induced inflammatory responses in astrocytes (Gupta et al., 2012[12]). Moreover, oleic acid is able to reverse the saturated fatty acid palmitate-induced insulin resistance and inflammation in skeletal muscle cells (Coll et al., 2008[10]). Accordingly, we expected the SFEIO, which contained unsaturated fatty acids, to exhibit anti-inflammatory activities.

Macrophages play a key role in immune responses during inflammation. Activation of macrophages by LPS releases inflammatory mediators, including NO and PGE_2_, which are generated via the oxidation of the terminal guanidine nitrogen of L-arginine by iNOS and the conversion of arachidonic acid by COX-2 (Posadas et al., 2000[29]). Both NO and PGE_2_ are involved in the progression of inflammation and carcinogenesis (Claria, 2003[9]). IL-1β, IL-6, and TNF-α are some of the major pro-inflammatory cytokines, which are produced by monocytes and macrophages which play a crucial role in the progression of the initial steps in the inflammatory response (Tao et al., 2009[34]; Young et al., 2002[37]). Thus, reducing the levels of NO, PGE_2_, IL-1β, IL-6, and TNF-α could be an effective strategy to inhibit inflammation. Current results suggest that SFEIO could inhibit the production of NO and PGE2 by the suppression of iNOS and COX-2 expression. Additionally, SFEIO reduced the production of IL-1β, IL-6, and TNF-α in LPS-induced RAW 264.7 cells.

NF-κB-mediated regulation of pro-inflammatory gene expression is controlled by transferases belonging to the IκB family. The transcription factor NF-κB could regulate the expression of pro-inflammatory mediators, including COX-2, iNOS, IL-1β, IL-6, and TNF-α (Kim et al., 2010[16]; Rhee et al., 2007[30]). indicate that the degradation and phosphorylation of IκB, a cytoplasmic inhibitor of NF-κB, could induce inflammatory stimuli, resulting in the translocation of the activated p65/p50 heterodimer from the cytoplasm to the nucleus. Current results indicate that SFEIO suppression the LPS induced IκB-α phosphorylation and degradation, and the activation of NF-κB p50. These results reveal that the effects of SFEIO on inflammatory mediators and cytokine production are mediated via the inhibition of NF-κB pathway.

Additionally, SFEIO could attenuate LPS-stimulated phosphorylation of JNKs and ERK, whereas it did not affect p38 MAPK. MAPKs include ERKs, JNKs, and p38 kinase (Shao et al., 2013[32]), and the phosphorylation of these proteins promotes the pro-inflammatory cytokine production in LPS-induced macrophages (Kim et al., 2013[17]; Yoon et al., 2012[36]). In particular, activation of ERK and JNK could regulate the responses in LPS-stimulated macrophages, inducing increased pro-inflammatory cytokine and iNOS production (Ajizian et al., 1999[2]; Uto et al., 2005[35]). These results demonstrate that the anti-inflammatory effect of SFEIO could down-regulate LPS-stimulated phosphorylation of JNK and ERK in RAW 264.7 cells.

In conclusion, the present study revealed that SFEIO treatment inhibited LPS-stimulated NO and PGE_2_ production in macrophages by suppressing iNOS and COX-2 expression, respectively. SFEIO also significantly inhibited the release of IL-1β, IL-6, and TNF-α in LPS-stimulated RAW 264.7 cells. These effects occurred due to inhibition of NF-κB signaling and JNK and ERK phosphorylation. These findings indicate that SFEIO may have potential anti-inflammatory activity.

## Notes

Eun-Yi Ko and Weon-Jong Yoon contributed equally as first author.

Daekyung Kim and Kil-Nam Kim (Jeju Center, Korea Basic Science Institute (KBSI), Jeju 690-140, Republic of Korea; Tel: +82-64-800-4933, E-mail: knkim@kbsi.re.kr) contributed equally as corresponding author.

## Acknowledgement

This research was supported by the project (C36290) offered by the Korea Basic Science Institute. This study was supported by a research grant from the Marine Biotechnology Program funded by the Ministry of Oceans and Fisheries of Korean Government (PM59122), and was partially supported by a research grant funded by the Korea Institute of Ocean Science & Technology (PE99411).

## Conflict of interest

The authors declare no conflict of interest.

## Figures and Tables

**Table 1:**
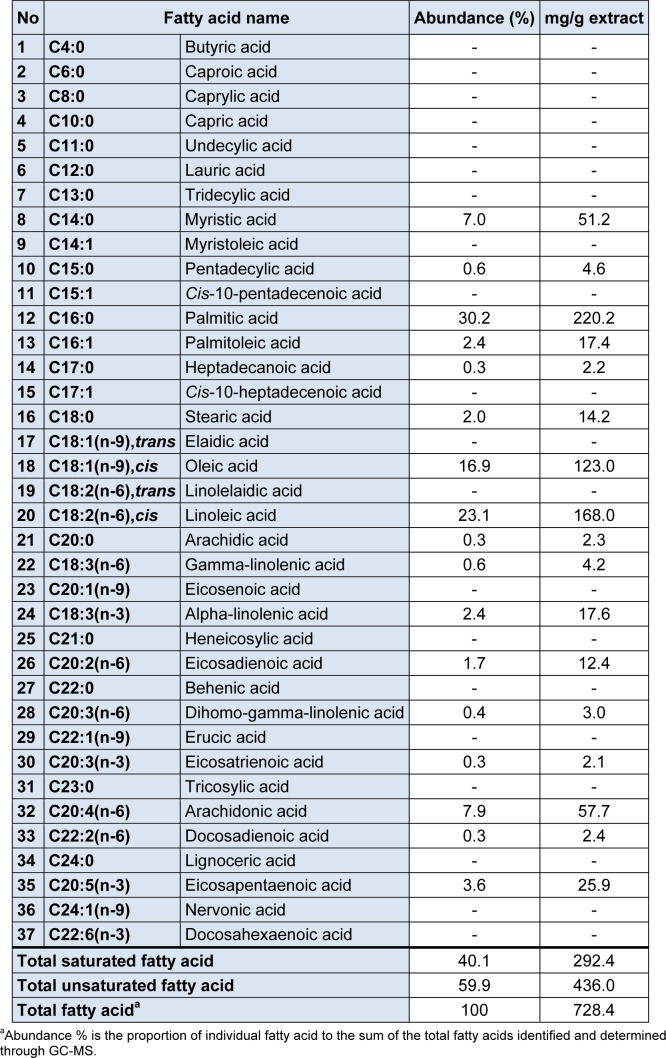
Fatty acid composition in SFEIO
